# Voxel-Wise Brain-Wide Functional Connectivity Abnormalities in Patients with Primary Blepharospasm at Rest

**DOI:** 10.1155/2021/6611703

**Published:** 2021-01-06

**Authors:** Pan Pan, Shubao Wei, Huabing Li, Yangpan Ou, Feng Liu, Wenyan Jiang, Wenmei Li, Yiwu Lei, Yanqing Tang, Wenbin Guo, Shuguang Luo

**Affiliations:** ^1^National Clinical Research Center for Mental Disorders, and Department of Psychiatry, The Second Xiangya Hospital of Central South University, Changsha, 410011 Hunan, China; ^2^Department of Neurology, The First Affiliated Hospital of Guangxi Medical University, Nanning, Guangxi 530021, China; ^3^Department of Radiology, The Second Xiangya Hospital of Central South University, Changsha, 410011 Hunan, China; ^4^Department of Radiology, Tianjin Medical University General Hospital, Tianjin 300000, China; ^5^Department of Psychiatry, The First Affiliated Hospital of China Medical University, Shenyang, Liaoning 110001, China; ^6^Department of Psychiatry, The Third People's Hospital of Foshan, Foshan, Guangdong 528000, China

## Abstract

**Background:**

Primary blepharospasm (BSP) is one of the most common focal dystonia and its pathophysiological mechanism remains unclear. An unbiased method was used in patients with BSP at rest to observe voxel-wise brain-wide functional connectivity (FC) changes.

**Method:**

A total of 48 subjects, including 24 untreated patients with BSP and 24 healthy controls, were recruited to undergo functional magnetic resonance imaging (fMRI). The method of global-brain FC (GFC) was adopted to analyze the resting-state fMRI data. We designed the support vector machine (SVM) method to determine whether GFC abnormalities could be utilized to distinguish the patients from the controls.

**Results:**

Relative to healthy controls, patients with BSP showed significantly decreased GFC in the bilateral superior medial prefrontal cortex/anterior cingulate cortex (MPFC/ACC) and increased GFC in the right postcentral gyrus/precentral gyrus/paracentral lobule, right superior frontal gyrus (SFG), and left paracentral lobule/supplement motor area (SMA), which were included in the default mode network (DMN) and sensorimotor network. SVM analysis showed that increased GFC values in the right postcentral gyrus/precentral gyrus/paracentral lobule could discriminate patients from controls with optimal accuracy, specificity, and sensitivity of 83.33%, 83.33%, and 83.33%, respectively.

**Conclusion:**

This study suggested that abnormal GFC in the brain areas associated with sensorimotor network and DMN might underlie the pathophysiology of BSP, which provided a new perspective to understand BSP. GFC in the right postcentral gyrus/precentral gyrus/paracentral lobule might be utilized as a latent biomarker to differentiate patients with BSP from controls.

## 1. Introduction

Primary blepharospasm (BSP) is one of the most common focal dystonia (FDT) in middle-aged and elderly individuals that is characterized by persistent or indirect excessive involuntary orbicularis oculi (pretarsal, preseptal, and periorbital portions) contraction [1] and blinking [2, 3] resulting in disability of daily activities. The patients have disabilities in reading, driving, eye contact, and even functional blindness as the disease progresses [4] which may cause social awkwardness and disastrous traffic accidents [5], seriously affecting their lives. Patients with BSP are associated with nonmotor manifestations except dyskinesia, including paresthesia such as dryness, photophobia, and mood disorders [6]. For patients with BSP, anxiety and depression that follow the onset might be worse than the illness itself [7].

In most cases, BSP starts in a sporadic state. A previous study suggested that dystonia was caused by abnormal brain remodeling and inhibition of circuits in motor areas [8]. The microstructure and functional alterations in multiple regions of the brain have been visualized following the continuous improvement of the magnetic resonance imaging (MRI) technology. It provides new insights into the pathophysiological mechanism of BSP and expands the traditional research focus.

In recent years, functional MRI (fMRI) has been recognized as an effective tool to study changes of brain function in patients with BSP. fMRI was initially used for task- or stimulation-based research in studies of brain function. However, the brain continued to engage in a large amount of spontaneous neural activity without performing any specific tasks. The participants were in the resting state which could reduce the complexity of task design and selective bias. Therefore, resting-state fMRI (rs-fMRI) bears more research and application significance.

Functional connectivity (FC) patterns in fMRI provide a novel representation for mental states because it can display dynamic behavior in the range of seconds and with an ample temporal-spatial structure [9]. As a key feature to establish the interaction and communication between different brain regions, FC in neural networks reflects the functional relationship between spatially separated cortical signals [10, 11]. FC can be extracted from different spatial scales such as within a specific region of interest (ROI) or at the whole-brain level [12]. Researchers have focused on FC between preselected brain areas via a ROI manner in several previous studies related to BSP [13]. These studies may miss crucial brain areas related to the critical pathological alterations of BSP, and the potential bias naturally exists in these studies due to the preselected ROIs. Voxel-based global-brain functional connectivity (GFC) analysis can be applied to investigate the pathophysiology of BSP to remedy this deficiency. GFC is helpful to obtain FC throughout the brain without a bias [14, 15]. At present, GFC is a metric that provides the connectivity of all voxels relative to all the other voxels and no prior seeds or network selection is required in the measure [14, 16]. We hypothesized that certain brain areas in patients with BSP would exhibit aberrant GFC, and then compared the imaging data between patients with BSP and healthy controls to elucidate the possible relationship to the mechanism of BSP by using GFC.

Patients with BSP are often accompanied by clinical manifestations of mental symptoms, especially depression and anxiety [17–19]. Therefore, all patients in this study were screened to determine whether they were accompanied with symptoms of anxiety or depression before evaluation. Anxiety and depression symptoms were assessed separately to manage for the hybrid effects of these symptoms by using Zung's Self-rating Anxiety Scales (SAS) [20] and Zung's Self-rating Depression Scales (SDS) [21]. The severity of BSP was assessed by using the Jankovic Rating Scale (JRS-S) [1]. Then, correlation analyses were conducted between variables such as the duration of disease or JRS-S scores and the GFC values in different brain regions to probe the correlation between clinical symptoms and GFC. Finally, we adopted the support vector machine (SVM) approach to detect whether the GFC values in relevant brain regions could identify patients from controls which was a manifestation of GFC as potential image biomarkers. SVM has been used in medical diagnosis and image processing as a supervisory machine learning technology. SVM is a computational algorithm that learns from experience and examples to assign labels to targets similar to other machine learning techniques. As an indirect measurement method, biomarkers could classify biological or pathogenic processes objectively, accurately, and reproducibly. Biomarkers in whatever form are extremely important for diagnosis, tracking disease progression, and clinical trials. MRI biomarkers have been widely studied in neuroscience and related fields due to the relative ease of use, cost-effectiveness, and noninvasivity [22].

## 2. Materials and Method

### 2.1. Subjects

A total of 26 patients with BSP were recruited from the Outpatient Department of Neurology in the First Affiliated Hospital of Guangxi Medical University. The inclusion criteria of the BSP group were as follows: (1) meeting the clinical guidelines of BSP for diagnostic criteria of BSP [23], (2) without other history of serious neuropathy or psychopathic disorders, (3) conventional MRI examination showed no structural lesion, and (4) no history of related drug therapy within 6 months prior to enrollment such as botulism toxin injection [24]. The excluded patient conditions were as follows: (1) not suitable for MRI scan, (2) secondary BSP caused by other diseases, and (3) family history of neuropathy or psychosis. General data of patients such as sex, age, course of the disease, and education level were collected after enrollment. All patients with BSP were examined by two professional neurologists and two psychiatrists. The neurologists were commissioned to use JRS-S to evaluate the severity of BSP. Meanwhile, psychiatrists assessed the severity of anxiety and depression in patients, respectively, using SAS and SDS.

A total of 24 healthy controls were also recruited from the local community. Healthy subjects were recruited as sex, age, and educational level matched with patients. The exclusion criteria of the healthy control group were (1) any history of serious neurological illness or psychiatric illness, (2) any family history of severe psychiatric or neurological diseases in the immediate family, (3) any surgical history and serious medical disease, and (4) conventional MRI scan revealed abnormal brain structure. All participants ranged in age from 18 to 60 years old and were right-handed.

### 2.2. Image Acquisition and Preprocessing

Detailed information about the image acquisition and preprocessing is presented in the supplementary file. Resting-state images were captured by a Siemens 3.0 T scanner. The DPABI software designed to process data automatically in Matlab [25] was used to preprocess the imaging data.

### 2.3. GFC Analysis

GFC was calculated as the average voxel-to-voxel connectivity throughout the brain [26, 27]. In Matlab, GFC was computed by averaging the correlations between the time series of each voxel and all other voxels within a gray matter of the entire brain [28]. Voxel was classified as gray matter by threshold setting (probability > 0.2), which was utilized to generated a gray matter mask in SPM8 [29]. The GFC was calculated as follows:

(1) $$\begin{array}{c} \text{GFC}=\overset{n}{\underset{b=1}{\sum }}\frac{r\left(T_{a,}T_{b}\right)}{n-1}, \end{array}$$

where the Pearson correlation coefficient (*r*) of time series Ts for a pair of given voxels *a* and *b* was calculated, and then, Fisher *r*-to-*z* transformation was used to convert the coefficients into *z* values [14, 16, 30]. The mean coefficient of a given voxel with all other voxels was calculated to obtain the GFC of this voxel. Two-sample *t*-tests were performed on the GFC spectrum between patients and controls. We calculated the frame-wise displacement (FD) value of each participant based on a past research [31]. The education level, sex, age, and mean FD were treated as uninterested covariates. The family-wise error (FWE) correction method was adopted to set the significance level at *p* < 0.05.

### 2.4. Statistical Analysis

We first extracted the mean *z* values of GFC from the brain clusters. After the normality of the conversion values was assessed, Pearson correlation analyses were performed between GFC and clinical variables such as illness duration, severity, and scores of SAS and SDS in the patients with BSP. The significance level was Bonferroni corrected at *p* < 0.05.

### 2.5. Using SVM for Classification Analysis

As a good learning classifier different from other machine learning technologies, SVM has been extensively used in classification problems for its ability to process high-dimensional data and high classification accuracy, especially suitable for the classification of small sample cases [32, 33]. It learns from experience and examples to assign labels to targets. The basic function of SVM is to separate binary-labeled data based on a beeline to maximize the distance between labeled data [34]. SVM is one of the famous classification techniques and usually provides the best classification for radiological computer-aided detection [35]. SVM analysis has the ability to reduce false positive in the estimation of abnormal lesions in the brain, with high accuracy, good mathematical processing, and intuitive geometric interpretation. SVM has a good accuracy even with small samples. Therefore, SVM is different from other machine learning technologies due to this advantage. As an auxiliary method for prediction, SVM has been widely used in the biomedical diagnosis [36]. The kernel function was used to help separate labeled data by SVM. One superiority of using kernel in SVM was that the kernel defined by SVM could be applied to nonvector inputs, which was particularly important in the medical field [34, 37, 38]. The possibility of abnormal GFC in these clusters was evaluated by SVM using the LIBSVM package [39] in MATLAB. The type of kernel used in this study was the default Gaussian kernel. The sample set was divided into a test set and a training set to observe the classification performance of target data. The hyperplane is selected to maximize the minimum distance from any data point to the hyperplane when the training data set presents a separable state [39]. A cluster of random SVM for classification and characteristic selection was established based on the brain fMRI data. A “leave-one-out” cross-validated method was adopted to optimize the parameters, and the most frequent features were selected to obtain a good sensitivity and specificity which were considered the most discriminative features and the most responsive markers [29, 40].

## 3. Result

### 3.1. Characteristics of the Participants

Due to excessive head movement, we excluded two patients from further analysis. The final sample consisted of 24 controls and 24 patients with BSP. The clinical variable characteristics of the participants are shown in Table 1. No significant differences were observed between patients with BSP and controls in age, sex, and level of education. Sensory tricks occurred in 19 patients with BSP (79.16%). In addition, 12 patients (50.00%) experienced more severe eyelid spasms while speaking. Moreover, a total of 3 patients with BSP (12.5%) were comorbid with depression.

### 3.2. Group Differences in GFC

**Figure 1:**
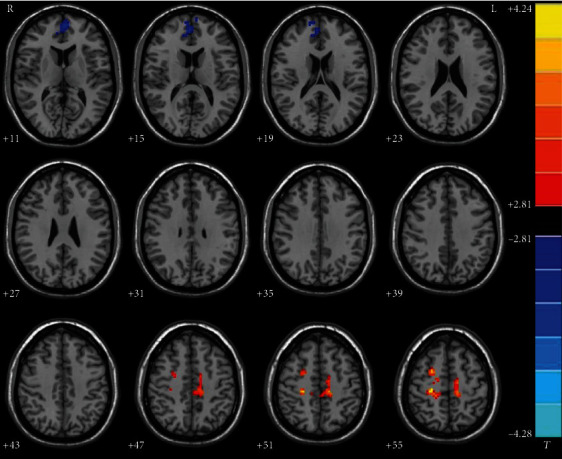
Abnormal GFC in patients with BSP relative to healthy controls. GFC: global-brain functional connectivity; BSP: primary blepharospasm.

Patients with BSP exhibited increased GFC in brain areas including the left paracentral lobule/supplement motor area (SMA), right superior frontal gyrus (SFG), and right postcentral gyrus/precentral gyrus/paracentral lobule compared with controls (Figure 1 and Table 2). The bilateral superior medial prefrontal cortex/anterior cingulate cortex (MPEC/ACC) of patients with BSP showed decreased GFC.

### 3.3. Correlation Analysis

There was no significant correlation between the GFC values in any aberrant brain areas and the severity of symptom, illness duration, and scores of SAS or SDS in the patients (*p* > 0.05).

### 3.4. SVM for Classification Analysis

**Figure 2:**
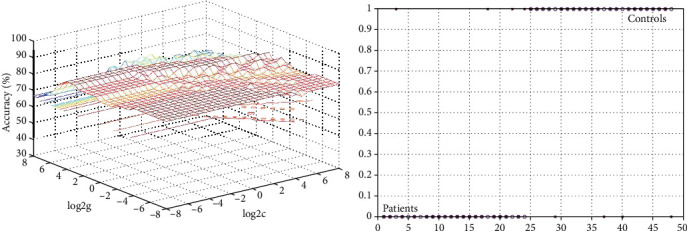
3D view of the classified accuracy with the best parameter using GFC in the right precentral gyrus/postcentral gyrus/paracentral lobule to differentiate the patients from the controls. The result was obtained in LIBSVM using a “leave-one-out” approach with default Gaussian kernel. GFC: global-brain functional connectivity.

**Figure 3:**
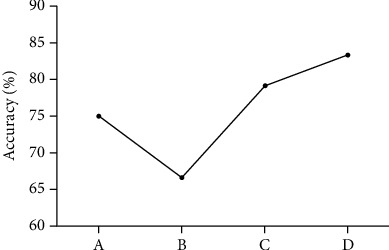
Visualization of classifications in SVM by using GFC in the different brain regions: (a) right SFG; (b) bilateral superior MPFC/ACC; (c) left paracentral lobule/SMA; (d) right precentral gyrus/postcentral gyrus/paracentral lobule. SVM: support vector machine; GFC: global-brain functional connectivity; SFG: superior frontal gyrus; MPFC: medial prefrontal cortex; ACC: anterior cingulate cortex; SMA: supplementary motor area.

We used accuracy, sensitivity, and specificity to quantify the performance of the SVM. The final sample in the present study included 24 patients and 24 controls. The SVM results indicated that 20 patients and 20 controls were correctly classified. Sensitivity was the ratio of the correctly classified patients to the total patients (20/24), whereas specificity was the ratio of the correctly classified controls to the total controls (also 20/24). Finally, the sum of the correctly classified patients and controls divided by the sum of the total patients and controls was the accuracy (40/48). The result showed that the GFC values in the right postcentral gyrus/precentral gyrus/paracentral lobule could discriminate patients from controls with optimal sensitivity, specificity, and accuracy (83.33%, 83.33%, and 83.33%, respectively) (Figure 2). The accuracies of other brain regions were unsatisfactory (Figure 3 and Table 3).

## 4. Discussion

In this study, rs-fMRI analysis was conducted by the GFC method to demonstrate changes across the whole-brain FC in patients with BSP. The BSP group exhibited significantly decreased GFC in the bilateral MPFC/ACC and increased GFC in the right SFG, which were included in the default mode network (DMN) compared with the controls. Meanwhile, the right postcentral gyrus/precentral gyrus/paracentral lobule and left paracentral lobule/SMA belonging to the sensorimotor network in patients with BSP showed increased GFC. Moreover, no significant correlation was observed between abnormal GFC in any brain area and the symptom severity, illness duration, and scores of SAS or SDS in the patients. The analysis of SVM suggested that the GFC values in the right postcentral gyrus/precentral gyrus/paracentral lobule could distinguish patients with BSP from controls with optimal accuracy and sensitivity.

Although dystonia has been traditionally regarded as a simple dyskinesia originating from the basal ganglia injury [41–43], the research trends on the pathological and physiological mechanism of dystonia have gradually shifted to the combination of sensorimotor [44]. The sensorimotor integration refers to the process of assisting motor program execution after sensory input signals that are integrated by the central nervous system. The abnormality of sensory-motor integration is an important mechanism of FDT. The sensory symptoms often precede motor symptoms in the process of FDT [6]. The patients often experience abnormal somatic sensations (such as discomfort and pain) that can be relieved by abnormal movements (sensory tricks) before the onset of motor symptoms. A previous study has exhibited patients with BSP accompanied by sensory tricks accounted for 81%~86% [45]. Sensory tricks occurred in the majority of the patients (79.16%) in the present study. These patients could temporarily alleviate their discomfort by making movements such as touching the cheeks, forehead, and underjaw or wearing glasses. This phenomenon related to a variety of sensorimotor processes, suggesting sensorimotor integration dysfunction in patients with BSP [2, 46].

The model of sensorimotor integration involved the coordination of high- and low-level nodes. SMA worked at a high level in the sensorimotor loop. It modulated the signals from low-level nodes and then projected them back to the primary motor cortex to calibrate the movement and motor execute commands with other high-level nodes [47, 48]. Abnormal function in SMA of patients with BSP has been reported in a previous study. The enhanced FC in SMA of patients with BSP may improperly encode sensory input information in the sensory-motor integration circuit. These misperceptions are more easily transmitted to the SMA, causing subsequent abnormal motor information to the primary motor area (M1) and leading to incorrect motor output or BSP [49]. Consistent with the previous study, increased GFC in SMA of patients with BSP may be associated with sensorimotor integration dysfunction during dystonia development in this study. We observed increased GFC values in the paracentral lobule in patients with BSP. The area extending from the precentral gyrus and postcentral gyrus that lies in the medial frontal cortex is the paracentral lobule [50]. The precentral gyrus and paracentral anterior lobe belong to M1, which can manage the localization of local movements of skeletal muscles. The precentral gyrus is closely related to the preparation or execution stages of movement. Functionally, the precentral gyrus receives projections from partial secondary somatosensory cortex of the postcentral gyrus which contains information of muscles and arthrosis, and converts behavioral commands into signals that encode various actions [51]. Functional anatomy suggests that SMA, precentral gyrus, and paracentral anterior lobe belong to the motor network. The postcentral gyrus, as the main region of the primary somatosensory cortex (S1), has been considered an important part of the pain network. Previous studies have shown that abnormal activation of the postcentral gyrus occurred in trigeminal neuralgia, postherpetic neuralgia, and other pain diseases ([52]; J. [53]). It suggests that the postcentral gyrus plays an important role in pain perception and intensity management in neuralgia [54]. The coordinated association of S1 and M1 was commonly defined as the sensorimotor cortex (SMC), which was mainly involved in motor control and somatosensory perception and pain processing [55, 56]. M1 plays an important role in controlling voluntary movement. The damage in M1 may be manifested as a reduction in peripheral inhibition, which results in excessive muscle contraction and unnecessary behavior [57]. Increased GFC in SMC of patients with BSP suggested that controlling eyelid movement required processing more complex information and procedures. Therefore, the SMC may act as an important role in the pathogenesis of BSP.

Bilateral superior MPFC/ACC and right SFG are the components of the DMN. The DMN was first proposed in 2001 that exhibited high levels of activity in resting state [58, 59]. The network mainly includes the MPFC, ventral ACC, posterior cingulate gyrus, dorsal thalamus, precuneus, hippocampus, inferior parietal lobule, and part of temporal lobes [60]. Previous studies have exhibited that MPFC is involved in a variety of higher brain functions including attention, motor preparation, execution, and inhibition [61, 62]. MPFC can send decision instructions to the motor or premotor areas to manage and control movement. Decreased GFC in this brain region may be associated with abnormal executive function in patients with BSP. The ACC can monitor ongoing target-directed behavior and provides signals in the event of response to conflict. Attention resources can also be efficiently allocated in the relevant brain regions according to the processing requirements of the current task [63]. Therefore, the ACC may play an advanced regulatory role in the DMN. The reduced GFC in the ACC indicated that the motor regulatory structure was destroyed in patients with BSP. The SFG, which belonged to the DMN, has been considered to be involved in certain cognitive and motor control tasks [64, 65]. The SFG is associated with some key nodes of the motor control network, such as the anterior central gyrus, caudate nucleus, and thalamus [66]. Alterative GFC in this area may partially contribute to dyskinesia in patients with BSP. Previous studies mainly focused on the correlation between abnormal FC in the SFG and the pathophysiological mechanism of mental disorder [67–69]. The specific function and related mechanism of BSP have not been conclusively determined. This result would extend this sight that SFG might be related to the dyskinesia of BSP.

There was no significant correlation between the GFC values and any clinical variables in the present study. The finding exhibited that emotional obstacle was not a direct response to FDT. Abnormal neural activity in these brain areas of patients with BSP may be an intrinsic trait alteration, independent of the illness duration, symptom severity, and symptoms of depression or anxiety, rather than secondary manifestations. Hence, patients with BSP may be accompanied with mood disorders that do not affect disease progression.

The result of SVM analyses showed that the GFC values in the right postcentral gyrus/precentral gyrus/paracentral lobule could differentiate patients with BSP from controls with high accuracy, specificity, and sensitivity of more than 0.8. This accuracy is especially satisfactory for the discrimination result, which was good for the establishment of diagnostic indicators. Thus, we inferred that increased GFC in the right postcentral gyrus/precentral gyrus/paracentral lobule could be utilized as a latent image biomarker to identify patients with BSP from healthy controls.

Traditional research methods which have focused on the FC alteration between predetermined brain areas by the ROI method may ignore the areas related to the core lesions in BSP. By contrast, the GFC approach adopted in this study was designed to explore whole-brain FC abnormalities. This method could detect potential FC changes and presented the results in an impartial manner. The present study was the first to explore whole-brain FC abnormal situation in patients with BSP. In addition, we adopted SVM analysis to determine whether GFC in the right postcentral gyrus/precentral gyrus/paracentral lobule could be utilized to distinguish between patients with BSP and controls. Finally, the fMRI data was acquired during the resting state rather than a specific task state. Hence, abnormal GFC may roughly reflect the pathological alterations of BSP.

Our study has several limitations. First, stratified analysis was not performed on the patients for different severity degrees and illness duration due to small sample size. Second, further assessment of other nonmotor performance (such as cognitive and sensory deficit) could be utilized to provide a comprehensive illustration in patients with BSP.

## 5. Conclusions

GFC has been considered appropriate in examining the differences in a large-scale functional organization of the brain because it provides a unique measure of whole-brain FC. Previous studies may miss crucial brain areas related to the critical pathological alterations of BSP [49, 70, 71], and the potential bias results naturally exist in these studies due to the preselected ROIs. The novelty of the present research is that we examined GFC abnormalities in patients with BSP, and the findings were reported in an unbiased way. The present study is the first to examine voxel-wise brain-wide FC in BSP, which indicates that abnormal GFC in brain areas associated with SMC and DMN might underlie the pathophysiology of BSP, which provided a new perspective to understand BSP. GFC in the right postcentral gyrus/precentral gyrus/paracentral lobule might be utilized as a latent biomarker to differentiate patients with BSP from controls.

## Figures and Tables

**Table 1:** Characteristics of the participants.

	Patients (*n* = 24)	Controls (*n* = 24)	*p* value
Sex (male/female)	8/16	6/18	0.53^a^
Age (years)	49.58 ± 8.58	50.88 ± 8.13	0.59^b^
Education (years)	10.38 ± 2.34	10.63 ± 2.16	0.70^b^
Illness duration (months)	10.00 ± 3.82		
Symptom severity	2.63 ± 0.82		
SAS	43.79 ± 8.11		
SDS	47.95 ± 8.58		

^a^The *p* value for sex distribution was obtained by a chi-squared test. ^b^The *p* values were obtained by independent-samples *t*-tests. SAS: Zung's Self-rating Anxiety Scales; SDS: Zung's Self-rating Depression Scales.

**Table 2:** Regions with abnormal GFC in the patients.

Cluster location	Peak (MNI)			Number of voxels	*T* value^a^
	*x*	*y*	*z*		
Right SFG	24	3	57	33	3.9932
Bilateral superior MPFC/ACC	-3	57	9	70	-3.7523
Left paracentral lobule/SMA	-12	-27	51	80	3.9892
Right precentral gyrus/postcentral gyrus/paracentral lobule	24	-27	54	75	4.2437

MNI: Montreal Neurological Institute; GFC: global-brain functional connectivity; MPFC: medial prefrontal cortex; ACC: anterior cingulate cortex; SMA: supplementary motor area; SFG: superior frontal gyrus. ^a^A positive/negative *T* value represents increased/decreased GFC in the patients relative to the controls.

**Table 3:** Differentiate the patients from the controls by GFC values in each brain region with the SVM method.

Brain regions	Accuracy	Sensitivity	Specificity
Right SFG	75.00% (36/48)	79.17% (19/24)	70.83% (17/24)
Bilateral superior MPFC/ACC	66.67% (32/48)	70.83% (17/24)	62.50% (15/24)
Left paracentral lobule/SMA	79.17% (38/48)	70.83% (17/24)	87.50% (21/24)
Right precentral	83.33% (40/48)	83.33% (20/24)	83.33% (20/24)
Gyrus/postcentral			
Gyrus/paracentral lobule			

GFC: global-brain functional connectivity; SVM: support vector machines; MPFC: medial prefrontal cortex; ACC: anterior cingulate cortex; SMA: supplementary motor area; SFG: superior frontal gyrus.

## Data Availability

The data that support the findings of this study are available from the corresponding authors upon request.

## Supplementary Materials

Supplementary Materials The supplementary material is the detailed process of image acquisition and preprocessing. This includes the scan sequence selection and the specific parameters to be set.
